# Supplementary material for: Cell Imaging Using Two-Photon Excited CdS Fluorescent Quantum Dots Working within the Biological Window
Source: Nanomaterials (Basel). 2019 Mar 5;9(3):369. doi: 10.3390/nano9030369 (PMC6474132; doi:10.3390/nano9030369)
Supplement: Supplementary file 1 [file nanomaterials-09-00369-s001.pdf]

Supplementary Materials

Cell Imaging Using Two-Photon Excited CdS Fluorescent Quantum Dots Working within the Biological Window

Nannan Zhang[1], Xiao Liu[1], Zhongchao Wei[1], Haiying Liu[1], Jie Peng[2,],*Liya Zhou[3], Hongmei Li[4], and Haihua Fan[1],*

1. Synthesis of CdS QDs

1.1. Reagents

The samples of $CdCl_2$•$2.5H_2O$ (99%), NaS•$9H_2O$ (99%), Thioglycolic acid (TGA), NaOH, Rhodamine 6G were purchased from Sig. Ma. Aldrich Company. Dulbecco's Modified Eagle Medium (DMEM), fetal bovine serum (FBS), Penicillin Streptomycin, trypsin-EDTA, phosphate buffer saline (PBS), 3-(4, 5-dimethylthiazol-2-yl)-2, 5-diphenyltetrazolium bromide (MTT) and Dimethyl Sulfoxide (DMSO) were purchased from Gibico. The experimental deionized water is obtained from a Milli-Q system (18.2 MΩ·cm, Millipore, France). None of reagents were further purified.

1.2. The preparation process of CdS QDs

100 mL of 0.02 mol/L $CdCl_2$•$2.5H_2O$ solution was loaded into a 250 mL the three-necked flask. 0.35 mL of Thioglycolic acid (TGA) was added to three-necked flask during magnetic stirring. The pH value of the mixed solution was adjusted to be 10.5 by the dropwise addition of 1 mol/L NaOH solution. Then 0.2459 g of NaS•$9H_2O$ was dissolved into 5 mL of deionized water and added to the above mixture. Finally, the solution mixture was heated to 100 ° C in an oil bath and refluxed for 5 hours before being cooled to room temperature. A transparent pale yellow CdS QDs solution was obtained.

2. The measurement of the photoluminescence quantum yields and the two-photon absorption cross-section of the CdS QDs solution

We calculated the photoluminescence quantum yields (PL QYs) and the two-photon absorption cross-section (σ_{2PA}) of the CdS QDs solution by the following equations (1) [1] and (2) [2], respectively. Rhodamine 6G (R6G) dissolved in methanol (QY=95%) was used as a reference [3]. In order to avoid reabsorption of the samples, the absorbance of the CdS QDs solution and the R6G solution were less than 0.1 at the same excitation wavelength [4].

The two-photon absorption cross-section (σ_{2cal}) of the R6G solution at excitation of 800 nm is 39.895 GM (1 GM=10-50 cm4•s•photon-1) [5].

$$QY_{sample} = \left(\frac{F_{sample}}{F_{ref}}\right)\left(\frac{A_{ref}}{A_{sample}}\right)\left(\frac{n^2_{sample}}{n^2_{ref}}\right) QY_{ref} \quad (1)$$

$$\sigma_2 = \sigma_{2cal} \times \frac{C_{cal}}{C} \times \frac{F}{F_{cal}} \times \frac{n_{cal}}{n} \times \frac{\eta_{cal}}{\eta} \quad (2)$$

In formula (1), F is the measured photoluminescence intensity, A is the absorbance at the excitation wavelength, and n is the refractive index of the solvent. In formula (2), C is the concentration of the sample, F is the two-photon luminescence intensity, and the η is the QY.

3. Experimental setup for the Two-photon luminescence

The experimental setup used for the two-photon excitation of CdS QDs is schematically shown in Figure S1.

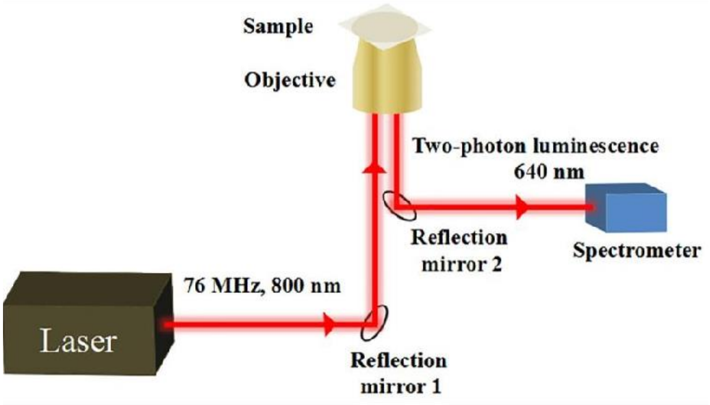

Figure S1. The experiment setup of two-photon excitation of CdS QDs

4. The upconversion luminescence lifetimes of CdS QDs

We measured the upconversion luminescence lifetimes of CdS QDs using a fluorescence lifetime spectrometer that is based on the technique of time correlated single photon counting (TCSPC). The result of measurement is shown in Figure S2.

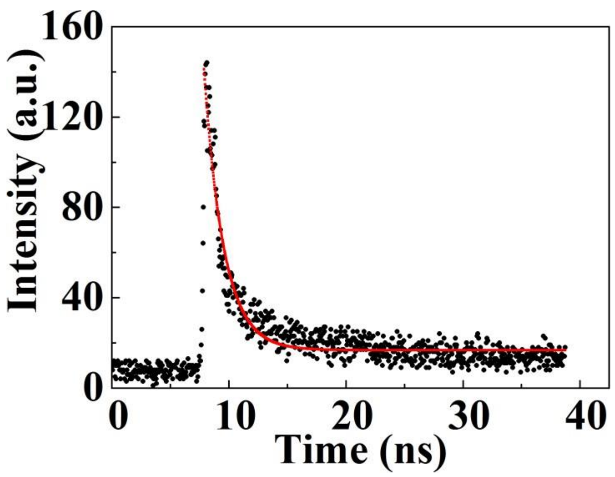

Figure S2. Two-photon excited luminescence decay curves (dots) of the CdS QDs solution and the best fitting curves (red line).

5. Cell Viability and Cellular Uptake of CdS QDs

The techniques of cell culture were similar to those described in reference [6]. Cell viability was measured using the MTT assay. The measurement was based on the method previously reported in reference [6]. The absorbance of the 96-well plate at 490 nm was measured using an iMark Microplate (BioRad). The cell viability was calculated by the following formula (3):

$$\text{Cell Viability} = (OD_{treated} - OD_{\text{blank}}) / (OD_{control} - OD_{blank}) \quad (3)$$

Where OD refers to the absorbance of each well. The subscripts "treated" and "control" refer to the HepG2 cells incubated with and without CdS QDs, respectively. The subscript "blank" refers to the cell medium in the absence of cells and CdS QDs. The morphology images of HepG2 cells incubated with CdS QDs at different concentrations are shown in Figure S3.

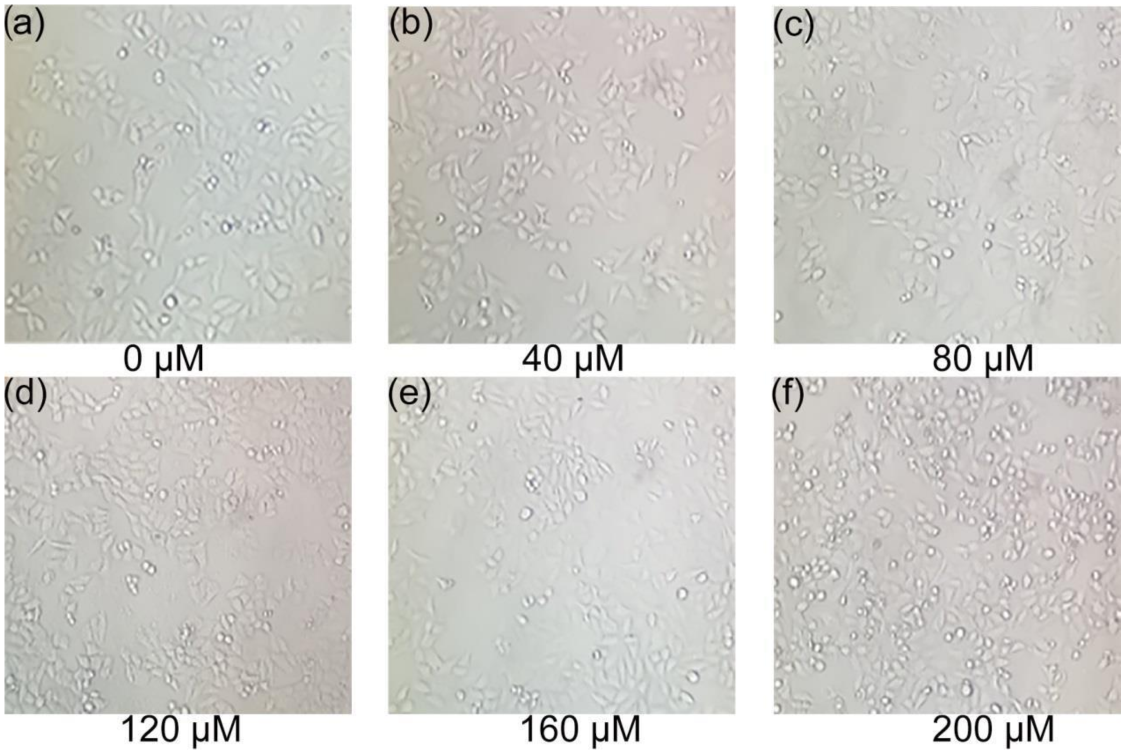

Figure S3. Morphology of HepG2 cells observed by using an inverted light microscope after incubating with CdS QDs at different concentration for 24 hours.

The uptake of CdS QDs of individual HepG2 cells was measured by inductively coupled plasma-mass spectrometry (ICP-MS) (ICAP-qc, Thermo Fisher, Germany) [7]. We incubated HepG2 cells with the CdS QDs in a humidified incubator (37 ° C, 5% CO_2) for 24 hours. Then, the cells were washed three times with Phosphate Buffered Solution (PBS) and centrifuged to form cell pellets. Finally, the cell pellets were fixed by glutaraldehyde (2.5%), embedded in resin, cut to ultra-thin slices, and stained by osmic acids. The samples were supported by Ni mesh coated with an ultra-thin carbon film. We obtained the distribution images and EDS data of CdS QDs in the cells using TEM (JEOL-1400HR, JEOL, Japan) equipped with energy-dispersive X-ray spectrometry (EDS) under an acceleration voltage of 200 kV. Figure S4 is the TEM image of a HepG2 cell incubated with CdS QDs for 24 hours.

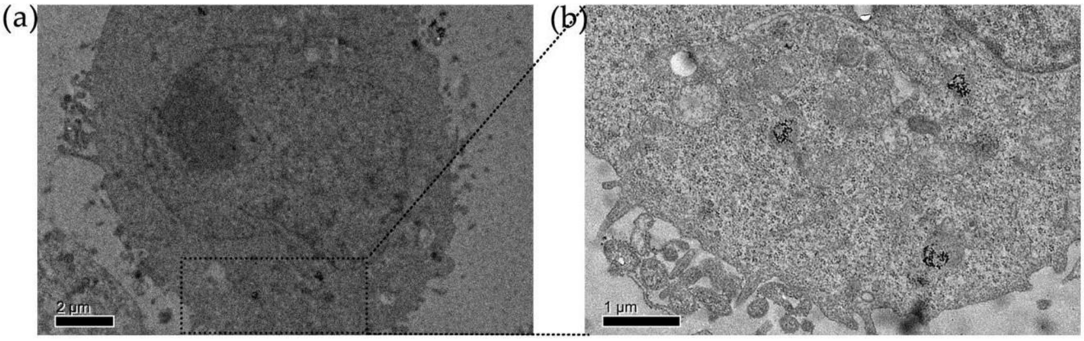

Figure S4. (a) TEM image of a HepG2 cell incubated with CdS QDs for 24 hours; (b) The enlarged TEM image of CdS QDs naturally created in the lysosome of the HepG2 cell.

6. The fluorescence brightness of cells

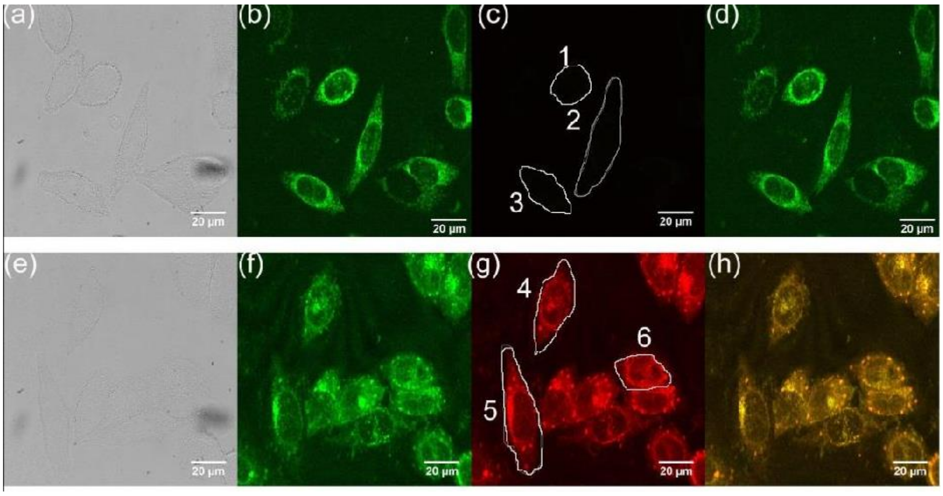

Figure S5. (a)-(d)The image of control group HepG2 cells without culturing with CdS QDs; (a) The bright field image; (b) the two-photon absorption fluorescence images with green channel; (c) red channel (d) the combination image of green and red channel; (e)-(h) The images of HepG2 cells incubated with CdS QDs for 6 hours; (e) The bright field image; (f) two-photon absorption fluorescence images with green channel; (g) red channel (h) combination image of green and red channel.

TableS1. The fluorescence brightness of the selected cells

Sample	(1)	(2)	(3)	(4)	(5)	(6)
**Area(cell)**	1	1	1	1	1	1
**Intensity(min)**	1	1	1	8	9	8
**Intensity(max)**	4	3	3	88	89	88
**Intensity(mean)**	1.8	1.0	1.6	35.3	38.2	39.4

References

1. Yang, W.M.; Zhang, B.; Zhang, Q.T.; Wang, L.X.; Song, B.; Ding, Y.; Wong, C.P. Adjusting the band structure and defects of ZnO quantum dots via tin doping. *Rsc Adv* **2017**, *7*, 11345-11354, 10.1039/c6ra25940e.
2. Xu, C.; Webb, W.W. Measurement of two-photon excitation cross sections of molecular fluorophores with data from 690 to 1050 nm. *J Opt Soc Am B* **1996**, *13*, 481-491, 10.1364/Josab.13.000481.
3. Schejn, A.; Fregnaux, M.; Commenge, J.M.; Balan, L.; Falk, L.; Schneider, R. Size-controlled synthesis of ZnO quantum dots in microreactors. *Nanotechnology* **2014**, *25*, 1-9,10.1088/0957-4484/25/14/145606.
4. Dhami, S.; Demello, A.J.; Rumbles, G.; Bishop, S.M.; Phillips, D.; Beeby, A. Phthalocyanine Fluorescence at High-Concentration - Dimers or Reabsorption Effect. *Photochem Photobiol* **1995**, *61*, 341-346, 10.1111/j.1751-1097.1995.tb08619.x.
5. Pu, S.C.; Yang, M.J.; Hsu, C.C.; Lai, C.W.; Hsieh, C.C.; Lin, S.H.; Cheng, Y.M.; Chou, P.T. The empirical correlation between size and two-photon absorption cross section of CdSe and CdTe quantum dots. *Small* **2006**, *2*, 1308-1313, 10.1002/smll.200600157.
6. Fan, H.H.; Le, Q.; Lan, S.; Liang, J.X.; Tie, S.L.;Xu, J.L. Modifying the mechanical properties of gold nanorods by copper doping and triggering their cytotoxicity with ultrasonic wave. *Colloid Surface B* **2018**, *163*, 47-54, 10.1016/j.colsurfb.2017.12.027.
7. Zhou, W.B.; Liu, X.S.; Ji, J. More efficient NIR photothermal therapeutic effect from intracellular heating modality than extracellular heating modality: an in vitro study. *J Nanopart Res* **2012**, *14*, 1-16, 10.1007/s11051-012-1128-6.
